# Primary Percutaneous Coronary Intervention of Acute Thrombotic Occlusion of a Low-Lying Tortuous Right Coronary Artery With a Multipurpose Catheter in an Octogenarian

**DOI:** 10.7759/cureus.46048

**Published:** 2023-09-27

**Authors:** Dibyasundar Mahanta, Shilpa Vinayak Gadade, Deepak Kumar Parhi, Debasish Das

**Affiliations:** 1 Department of Cardiology, SUM Hospital, Bhubaneswar, IND; 2 Department of Cardiology, All India Institute of Medical Sciences, Bhubaneswar, IND

**Keywords:** octogenarian, multipurpose catheter, low lying, right coronary artery (rca), primary percutaneous coronary intervention (pci)

## Abstract

Right coronary artery intervention is usually accomplished with a Judkins right (JR) coronary guide catheter. Abnormal right coronary artery take-off from the right coronary sinus poses difficulty in engaging the right coronary artery with a conventional JR guide catheter. We report a rare case of primary percutaneous intervention of the right coronary artery which was performed with a multipurpose catheter as the patient had an extremely low-lying coronary artery from the coronary sinus where conventional catheters could not engage the right coronary artery in an octogenarian with acute inferior wall ST-elevation myocardial infarction in cardiogenic shock.

## Introduction

The right coronary artery during the percutaneous coronary intervention (PCI) is usually engaged with a Judkins right (JR) guide catheter 6F 3.5 [1]. The difficulty arises when there is a congenital anomalous origin of the right coronary artery from the right coronary sinus. The origin of the right coronary artery can be classified as high, normal, or low from the coronary sinus. High origin of the right coronary artery from the coronary sinus can be engaged with JR, Amplatz left (AL), and Amplatz right (AR) coronary catheters [1]. The difficulty arises when the right coronary artery arises extremely low from the right coronary sinus and almost lies at the bottom of the coronary sinus when conventional catheters including tiger, JR, Judkins left (JL), AL, and AR catheter fail to engage the ostium. We describe a rare case of primary percutaneous coronary intervention (PPCI) of an extremely low-lying right coronary artery from the right coronary sinus with a multipurpose catheter (MPA) in an octogenarian having acute inferior wall ST-elevation myocardial infarction in cardiogenic shock. 

## Case presentation

**Figure 1:**
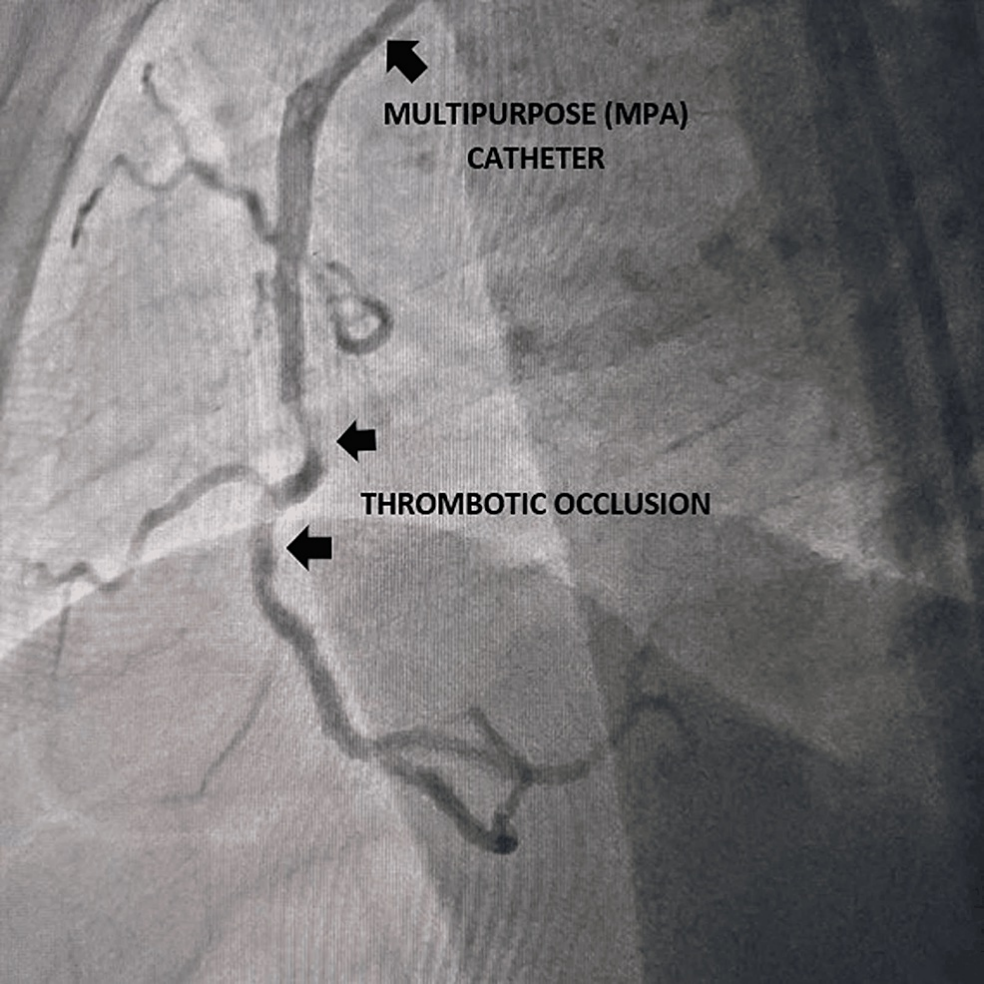
Down-pointing multipurpose catheter (MPA) engaging the low-lying right coronary artery

**Figure 2:**
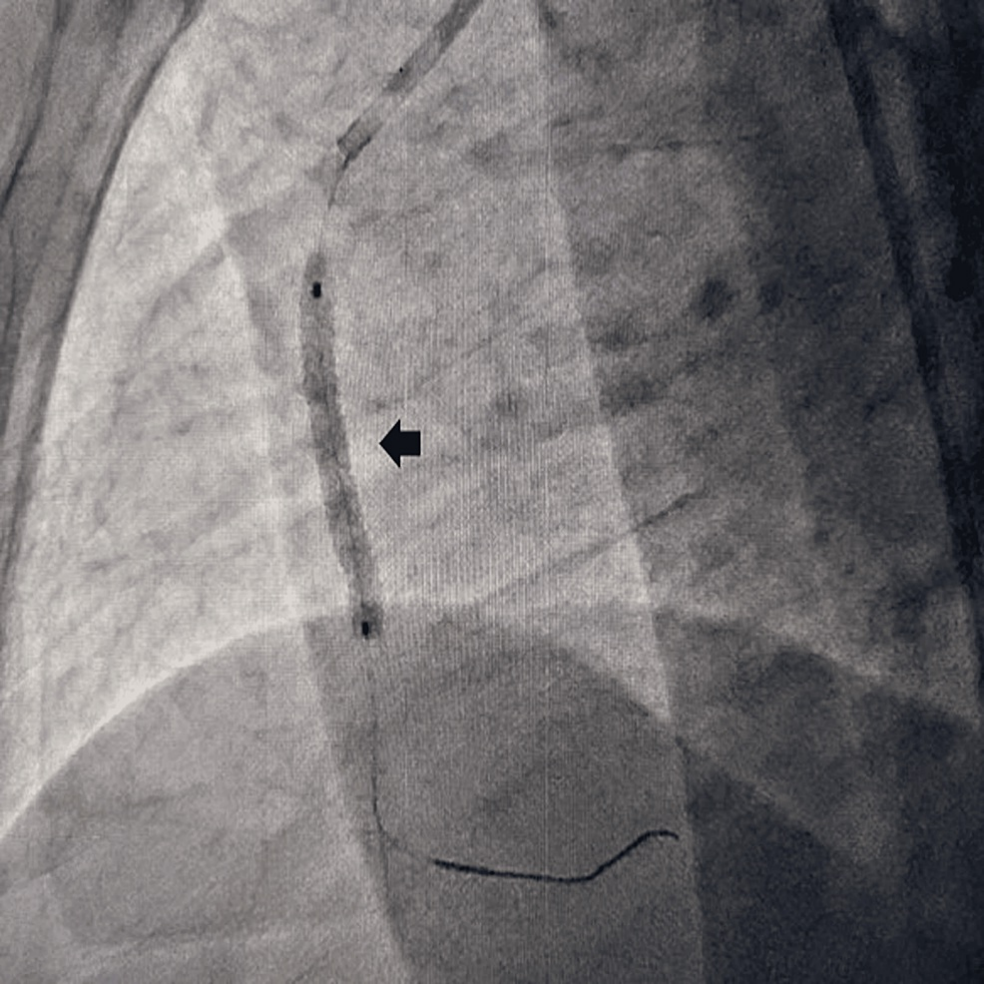
3x28 mm Drug-eluting stent deployment across the lesion

**Figure 3:**
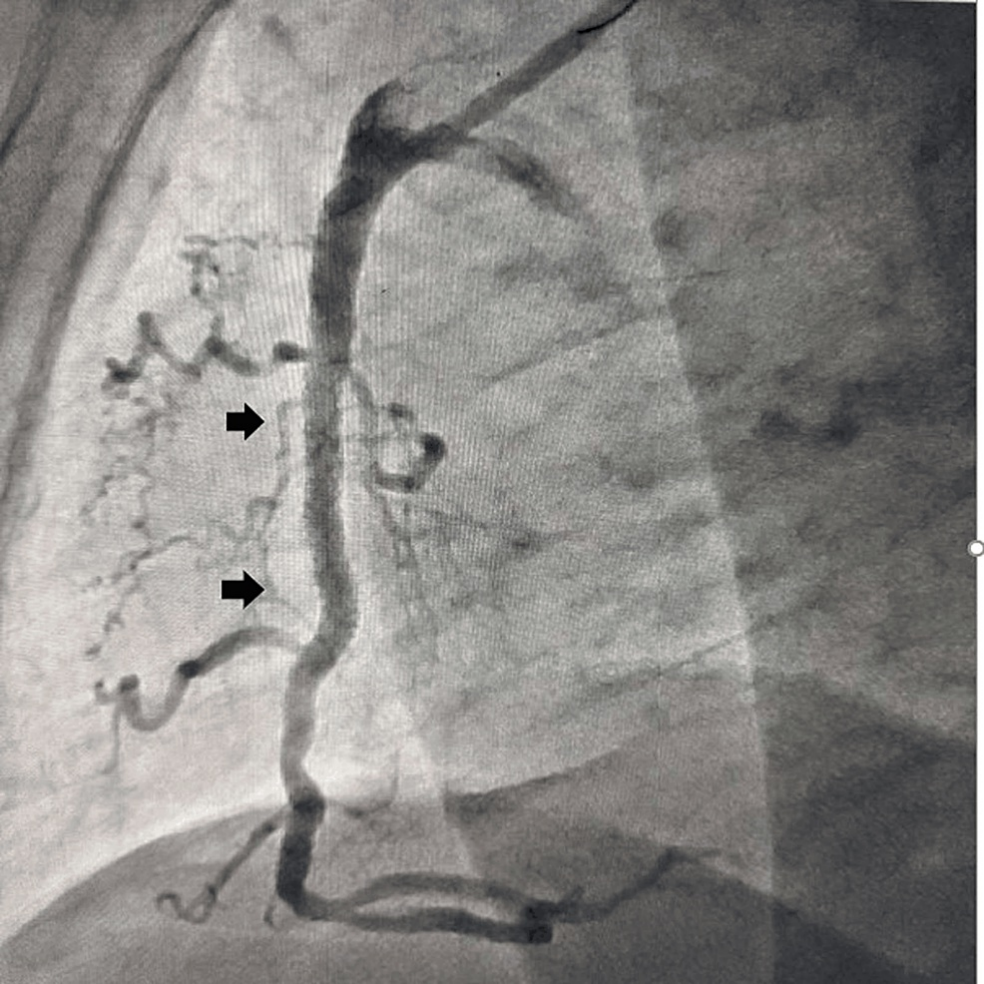
After coronary angioplasty, good angiographic result with TIMI III flow was achieved TIMI III, Thrombolysis In Myocardial Infarction grade 3.

An 84-year-old female presented to the emergency department with acute-onset retrosternal chest discomfort with diaphoresis and shortness of breath for the last hour without any palpitation, presyncope, or syncope. She has been diabetic and hypertensive for the last 30 years on oral hypoglycemic agents and angiotensin receptor blockers. She had no history of effort angina in the past, but her exercise capacity was limited due to her elderly age. She had a blood pressure of 80/40 mm Hg in the right arm supine position and she had a heart rate of 56 beats per minute. She had ongoing angina with oxygen saturation (SpO_2_) of 94% in room air. Baseline electrocardiogram (EKG) revealed ST elevation in inferior leads (II, III, aVF) with reciprocal ST depression in aVL and anterior leads (V1-4). Echocardiography revealed regional wall motion abnormality in the inferior posterior wall with left ventricular mild systolic dysfunction with an ejection fraction (EF) of 42% and mild ischemic mitral regurgitation. As she was in cardiogenic shock, she was infused with 1 liter of normal saline for over 1 hour, noradrenaline infusion was started and was rushed immediately to the cath lab for PPCI. Injection of the coronaries revealed a normal left coronary system with acute subtotal thrombotic occlusion of the right coronary artery. Interestingly, the right coronary artery was extremely low-lying in nature almost originating from the bottom of the coronary sinus. Right coronary ostium could not be engaged with conventional JR, AL, AR, and Ikari guide catheters. As the coronary ostium was low-lying almost in the bottom of the coronary sinus, we engaged it with a 6F MPA with a down-pointing tip which effectively engaged and provided good support during coronary intervention (Figure 1). The lesion was crossed with 0.014'' run-through guide wire, predilated with a 2x15 mm semi-compliant balloon, and stented with a 3x28 mm drug-eluting stent (Figure 2), which achieved good angiographic result with TIMI III (Thrombolysis In Myocardial Infarction grade 3) flow (Figure 3). The patient became hemodynamically stable after the procedure, and was put on dual antiplatelets aspirin and ticagrelor, low-dose beta-blocker, moderate-dose statin, and low-molecular-weight heparin in view of extremely elderly age. She was discharged in stable condition on the third postoperative day without any periprocedural complication. Our case is an interesting illustration of the use of an MPA in cases of PPCI of the right coronary artery where conventional catheters fail to engage and it effectively engages and also provides good support during the intervention of even tortuous right coronary artery.

## Discussion

Anomalous origin of the right coronary artery is most often engaged with AL, AR, and Ikari catheters [2]. However, the Amplatz group of guide catheters is more prone to cause ostial injury during coronary intervention. Anomalous origin of the right coronary artery has been engaged with a tiger catheter through the femoral route [3]. We report a rare case of engaging an anomalous right coronary artery arising from the bottom of the right coronary sinus with an MPA which effectively engaged the right coronary artery with good support for successful coronary intervention in an octogenarian with cardiogenic shock. The only disadvantage of engaging the right coronary artery with an MPA is that it may cause injury to the coronary intima during intervention with its down-pointing soft tip [4]. The right coronary artery with upper take-off is usually engaged with an AL or AR guide catheter. Sometimes right coronary artery harbors significant ostial disease which requires air mailing of the guide wire first and subsequent engagement of the guide catheter. Myocardial infarction patients in cardiogenic shock require urgent and swift coronary intervention and the selection of a proper guide catheter with rapid engagement of the guide catheter to the coronary ostium with subsequent swift coronary intervention is the key step in achieving successful coronary intervention. 

## Conclusions

We present a rare case of PPCI in a low-lying tortuous right coronary artery where an MPA effectively engaged the right coronary artery with good support and resulted in successful coronary intervention in an octogenarian with cardiogenic shock. The use of an MPA can be life-saving during the intervention in a low-lying right coronary artery from the coronary sinus. 

## References

[1] Di Mario C, Sutaria N (2005). Coronary angiography in the angioplasty era: projections with a meaning. Heart.

[2] Ben-Dor I, Weissman G, Rogers T (2021). Catheter selection and angiographic views for anomalous coronary arteries: a practical guide. JACC Cardiovasc Interv.

[3] Datta G, Rai DP (2016). Abnormal origin of right coronary artery and use of Tiger catheter through femoral route. Indian Heart J.

[4] Kindya B, Lisko J, Inci E, Khatri J, Nicholson W, King S (2022). Left heart catheterization using the single catheter radial approach with the multipurpose catheter: teaching an old dog new tricks. Clin Cardiol.

